# Rendering Banana Plant Residues into a Potentially Commercial Byproduct by Doping Cellulose Films with Phenolic Compounds

**DOI:** 10.3390/polym13050843

**Published:** 2021-03-09

**Authors:** Rosa E. A. Nascimento, Joana Monte, Mafalda Cadima, Vítor D. Alves, Luísa A. Neves

**Affiliations:** 1 LAQV/REQUIMTE, Chemistry Department, Faculdade de Ciências e Tecnologia, Universidade Nova de Lisboa, Campus de Caparica, 2829-516 Caparica, Portugal; re.nascimento@campus.fct.unl.pt (R.E.A.N.); joana.monte@campus.fct.unl.pt (J.M.); mafaldacadima@gmail.com (M.C.); 2 LEAF, Linking Landscape, Environment, Agriculture and Food, Instituto Superior de Agronomia, Universidade de Lisboa, Tapada da Ajuda, 1349-017 Lisbon, Portugal; vitoralves@isa.ulisboa.pt

**Keywords:** banana plant, cellulose, films, leaves, phenolic compounds, pseudostem

## Abstract

This study seeks to render residues from banana plants into a useful byproduct with possible applications in wound dressings and food packaging. Films based on cellulose extracted from banana plant pseudostem and doped with phenolic compounds extracted from banana plant leaves were developed. The phenolic compounds were extracted using batch solid-liquid and Soxhlet methods, with different drying temperatures and periods of time. The total phenolic content and antioxidant activity were quantified. The optimum values were obtained using a three-day period batch-solid extraction at 40 °C (791.74 ± 43.75 mg/L). SEM analysis indicates that the pseudostem (PS) films have a porous structure, as opposed to hydroxyethyl cellulose (HEC) films which presented a homogeneous and dense surface. Mechanical properties confirmed the poor robustness of PS films. By contrast HEC films manifested improved tensile strength at low levels of water activity. FTIR spectroscopy reinforced the need to improve the cellulose extraction process, the success of lignin and hemicellulose removal, and the presence of phenolic compounds. XRD, TGA and contact angle analysis showed similar results for both films, with an amorphous structure, thermal stability and hydrophilic behavior.

## 1. Introduction

Throughout human history, plants have served as a source of healing and for maintaining health [1]. Around 80% of developing countries use plants as medicines. However, there is a paucity of literature cataloguing the chemical constituents of plants and their potential in medicine. In recent years, some studies have been conducted to catalogue the chemical constituents of different plants with a view to better understanding their properties, safety and efficacy [2,3].

This is the context of our study, which focuses on the banana plant, *Musa acuminata* Colla, from the island of Madeira, Portugal. The banana plant has a rhizome, the stem, which grows horizontally underground and a pseudostem formed by an apical meristem from which grows the spirally arranged leaves [4]. The banana plant is the largest herbaceous plant; its pseudostem reaching a height of up to 10^–15^ m in some wild species. It also has the largest leaf area, varying from 1.27–2.80 m^2^/leaf, depending on the growing conditions [5]. The time from plantation to bunch harvesting ranges from 8 to 13 months [6].

There are at least 200–300 banana clone varieties in the world. Banana is the cheapest and most popular fruit throughout tropical and subtropical regions of the world [6]. The average global banana production in 2017–2019 reached 116 million tonnes [7]. Madeira and the Azores islands are the only places in Portugal where bananas are produced commercially. During 2020, the banana production on the island of Madeira increased by 29.2%, resulting in the sale of almost 23,000 tons [8]. Despite large banana production worldwide, only 12 wt% of banana plants are used, generating some 220 tons of banana residue per hectare of crop. These residues consist mainly of lignocellulosic material. They are discarded by farmers into rivers, lakes and on roadsides, causing serious environmental problems [9].

Phytochemicals, especially phenolic compounds on fruits and vegetables, are the main bioactive compounds known to have health benefits [10]. The diversity of phytochemical compounds in the banana plant provides potential for considerable phytochemical and pharmacological studies. The development of an allopathic or phytomedicine from *Musa* residues depends on a detailed investigation around the issues of quality control, efficacy, safety, toxicity and ethnopharmacological research, taking into account the fact that these phytochemical compounds are very sensitive to genetic and environmental factors, farming practices, fruit maturity stage at harvest, harvesting time, post-harvest handling and processing, and storage conditions [11,12].

Free radicals are molecular fragments but fortunately, oxidation may be counteracted by the consumption of antioxidants such as polyphenols, carotenoids and ascorbic acid (vitamin C) [13,14]. Natural antioxidants are preferable to synthetic antioxidants by virtue of being free of the toxicity and health hazards present in the latter [15,16]. They benefit from their anti-inflammatory, antiallergenic, antibacterial and antithrombic properties [10]. The banana contains a high number of phenolic compounds, mainly in the pulp, varying between 30–60 g phenolic compounds/100 g of fresh pulp, while the phenols on the banana peel varies between 0.90–3.0 g/100 g of dried peel [17,18].

As mentioned above, banana crops include huge amounts of residues with no economic value. This ‘waste’ may have considerable environmental impact and this study aims to analyze these residues to understand which compounds of interest could be extracted from them. After extracting these compounds, characterize them to understand which possible applications they can adapt to (such as wound dressing or packaging, for example), thereby reducing some of this deleterious impact and potentially adding value to the local economy.

The process starts with the extraction of the phenolic compounds from the banana plant leaves, using different extraction methods (batch solid-liquid and Soxhlet) and a range of experimental conditions including: drying temperature of the leaves (20, 40, 50 and 60 °C) and time period extraction (1–5 days), in order to understand which method produces the optimum result. The Total Phenolic Content (using the Folin Ciocalteau method) and antioxidant activity (using 2,2-diphenyl-1-picrylhydrazyl method) of the extracts were quantified. Cellulose was extracted from banana plant pseudostem and quantified by high pressure liquid chromatography (HPLC). These extracts were used to prepare films which were analyzed by the following techniques: scanning electron microscopy (SEM), X-ray diffraction (XRD), thermogravimetric analysis (TGA), Fourier transform infrared (FTIR) spectroscopy, calculated contact angle and mechanical properties. 

## 2. Materials and Methods

### 2.1. Materials

Banana plant leaves and pseudostem were provided by a farmer on the island of Madeira (an autonomous region of Portugal). All the samples were harvested from the same cultivation area and were therefore exposed to the same cultivation conditions. The material was stored at −20 °C prior to use.

### 2.2. Methods

#### 2.2.1. Phenolic Compounds Extraction

The phenolic compounds were extracted from the leaves of the plant using methanol (≥99.9%, Sigma-Aldrich, Darmstadt, Germany) as an extraction solvent. Methanol is recommended as a solvent for the extraction of relevant phytochemicals [19]. The samples were cleaned with cold tap water, dried with paper and then cut into small pieces (2 × 2 cm^2^). They were then dried in a cabinet dryer (Venticell, MMM Group, Planegg, Germany) for 7 h, with 2.0 m/s airflow using four different temperatures: 20, 40, 50 and 60 °C. The maximum drying time was 7 h, as more drying time results in the loss of antioxidant compounds. The highest temperature used was 60 °C as higher temperatures will result in the loss of around 90% of antioxidant activity [20]. The phenolic compounds were extracted using two different methods: batch solid-liquid and Soxhlet.

##### 2.2.1.1. Batch Solid-Liquid Extraction

The extraction of phenolic compounds using a methanol batch solid-liquid extraction method was performed in accordance with the work of Sagrin et al. [21] with slight modifications. Five grams of dried sample were crushed to powder using a crushing machine (A10, IKA, Staufen, Germany). Then, 100 mL of methanol was added for 3 days at room temperature. The sample was filtrated using a filter paper (185 mm, FILTER-LAB, Barcelona, Spain) and then concentrated using a rotatory evaporator (R-210, Buchi, Flawil, Switzerland), at 40 °C and 300 rpm. The sample was then stored at 4 °C until further analysis.

##### 2.2.1.2. Soxhlet Extraction

Sample powder (15.6 g) was added to the Soxhlet extraction set-up. Then, 250 mL of methanol (≥99.9%, Sigma-Aldrich) were added to the extraction flask. The extraction was performed for 7 h followed by filtration, concentration and storage, as described above in Section 2.2.1.1.

##### 2.2.1.3. Extracts Analysis

Determining Total Phenolic Content (TPC) Using Folin Ciocalteau Method

The Total Phenolic Content (TPC) was determined in accordance with the method used by Lim et al. [14], with some adjustments. Sodium carbonate solution was prepared by mixing 200 g sodium carbonate (≥99.0%, PanReac AppliChem, Darmstadt, Germany) with 800 mL deionized water, followed by 1 h sonication (DT 106, Bandelin Electronic, Berlin, Germany) and filtration (185 mm, FILTER-LAB). The calibration curve was performed, using 2500 mg/L gallic acid (99.0%, Sigma-Aldrich) samples with a dilution ratio of 1:1 of water:gallic acid. 

The analyzed samples, containing 1.58 mL of deionized water, 20 μL extract and 100 μL Folin reagent (PanReac AppliChem), were submitted to 5 sec vortexing (Reax 2000, Heidolph, Schwabach, Germany). Afterwards, 300 μL sodium carbonate were added and the samples were placed in the digestor (SBH130D, Stuart, Staffordshire, UK) for 30 min at 40 °C. The absorbance was measured in an UV/VIS spectrometer (Evolution 201, Thermo Fisher Scientific, Waltham, MA, USA), at a wavelength of 765 nm. The TPC was quantified using calibration curves established with pure gallic acid standards and expressed as mg/L.

Antioxidant Activity Determination by DPPH Method

Antioxidant activity was measured using the Brand-Williams et al. [22] method, with some modifications. The 2,2-diphenyl-1-picrylhydrazyl (DPPH) method is a colorimetric method that evaluates the in vitro antioxidant properties by mixing the sample with DPPH solution and then recording the absorbance after a defined period of time. The DPPH stock solution was prepared with 12 mg DPPH (95.0%, Alfa Aesar, Haverhill, MA, USA) and 100 mL methanol (≥99.9%, Sigma-Aldrich). The working solution was prepared by diluting the DPPH stock solution in methanol with a ratio of 1:5, to obtain a solution of 60 μM DPPH. The samples were prepared as described in Table S1—Supplementary Material.

The solutions were kept in the dark for 30 min at room temperature, until the reaction was detected. To analyze the antioxidant activity of the banana plant leaf extracts, the absorbance of the samples prepared were measured using a UV/VIS spectrometer (Evolution 201, Thermo Fisher Scientific) at the wavelength of 515 nm [23]. This wavelength corresponds to the DPPH reduced form peak absorbance. In the presence of antioxidant compounds, the DPPH’s free radical is captured by the antioxidant compound present in the solution, leading to the reduction of DPPH and, consequently, the colour solution changes from purple to yellow. The intensity of the yellow colour of the solution is directly proportional to the antioxidant activity of the sample and to the DPPH radical scavenging effect (%) [15]. The higher the percentage of DPPH, the stronger the antioxidant activity, meaning a high availability of hydrogen donation. DPPH inhibition percentage was calculated using Equation (1):

(1) $$\%\;\mathrm{DPPH}\;\mathrm{inhibition}=\frac{{\mathrm{Abs}}_{\mathrm{white}}-[({\mathrm{Abs}}_{\mathrm{extract}}-{\;\mathrm{Abs}}_{\mathrm{control}})\times \mathrm{Dilution}\;\mathrm{Factor})]}{{\mathrm{Abs}}_{\mathrm{white}}}$$

where % DPPH inhibition is the DPPH inhibition percentage (%); Abs_white_ the white solution’s (DPPH) absorbance; Abs_extract_ the extract solution’s absorbance; Abs_control_ the control’s absorbance; and Dilution Factor the dilution used in the analyzed samples.

#### 2.2.2. Cellulose Extraction and Analysis

Cellulose extraction was carried out following the Prado et al. [24] method, with some adjustments [23]. This procedure includes four steps:

(1) Fiber preparation: banana plant pseudostem was cut into small pieces (2 × 2 cm) and immersed in continually stirred deionized water, at 80 °C for 1 h. This enables the removal of the non-cellulosic compounds. Fibers were washed in tap water, to remove soluble sugars and impurities and then dried at 100 °C for 3 h in a cabinet dryer (Venticell, MMM Group, München, Germany).
(2) Fibers mercerization: 1 g fibers were treated with 20 mL 5% NaOH (40%, AkzoNobel Fine Chemicals, Amsterdam, The Netherlands), at 90 °C for 1 h, under stirring. Afterwards, samples were cooled at room temperature, filtrated and washed with deionized water, until pH = 7. The fibers were then dried, at 60 °C, in a cabinet dryer (Venticell, MMM Group), until a constant mass value was reached.
(3) Fibers bleaching: 1 g of fibers were treated with 40% [16% (*v*/*v*) H_2_O_2_ (9.0%, Alifar) + 5% NaOH (40%, AkzoNobel Fine Chemicals, Amsterdam, The Netherlands)], at 55 °C, for 90 min, under stirring. This step allows the removal of residual hemicellulose and lignin. The mixture was cooled at room temperature and filtered under vacuum. The resulting fibers were washed with deionized water until pH = 5 and dried at 60 °C, until constant mass was reached.
(4) Acid-catalyzed hydrolysis: dried pseudostem fibers were slowly added into the 60 wt% H_2_SO_4_ (95.0–97.0%, Honeywell, Charlotte, NC, USA) acid solution, previously cooled in an ice bath, under vigorous stirring and heated until 45 °C, for 1 h. To stop the reaction, the mixture was immediately cooled in an ice bath and 500 mL deionized ice water was added. The addition of deionized water dilutes the acid solution, resulting in a turbid supernatant. The resulting solution was dialyzed (12.0 S, Carl ROTH, Karlsruhe, Germany) against deionized water until pH = 7 was reached. This step allows the removal of free acid molecules from the suspension. Afterwards, the neutral solution was lyophilized (CoolSafe, ScanVac, Allerod, Denmark) and stored in a desiccator.

To confirm the success of cellulose extraction from banana plant pseudostem, 5 mg of the extract was digested with 5 mL 5% H_2_SO_4_ (95.0–97.0%, Honeywell), at 100 °C for 2 h. Glucose identification and quantification was done using high pressure liquid chromatography (HPLC) (Infinity 1100, Agilent Technologies, Santa Clara, CA, USA), and comparing its retention time with the glucose standard (purity ≥ 99.5%, Sigma-Aldrich), using a differential refractive index detector. The analysis was done using a CarboPac PA10 (4 × 250 mm^2^) analytical column. A gradient elution of 18 mM NaOH (40%, AkzoNobel Fine Chemicals) was performed using the flow rate of 1 mL/min, injection volume of 10 μL and the column was maintained at 25 °C.

#### 2.2.3. Films Development

##### 2.2.3.1. Cellulose-Based Films

The preparation of cellulose-based films was carried out based on the evaporation method of Lim et al. [25], with some variations. Commercial hydroxyethyl cellulose (HEC) (≥85.0%, Sigma-Aldrich) was used to compare the results with those obtained by extracting the cellulose from the banana plant pseudostem (PS).

Cellulose of HEC or PS (0.2 g) were weighed and added into 5 mL of deionized water. The resulting mixture was stirred on a heating and stirring plate (Arex CerAITopTM, Velp Scientifica, Usmate Velate, Italy) at 300 rpm and 50 °C for 60 min. Afterwards, the solution was centrifuged (4-16KS, Sigma-Aldrich) to degas, at 10,000 rpm for 5 min. The solution was then poured into a Teflon petri dish and left to dry in a cabinet dryer (Venticell, MMM Group), at 60 °C for 12 h.

In order to further understand the impact of water vapour on the films’ properties, the samples were left uncovered and exposed to atmospheres with different relative humidity values, at 30 °C, by using saturated solutions of potassium chloride (KCl) (≥99.0%, Sigma-Aldrich)—a_w_ of 0.843—, sodium chloride (NaCl) (≥99.5%, PanReac AppliChem)—a_w_ of 0.753—and sodium bromide (NaBr) (≥98.5%, ITW Reagents) —a_w_ of 0.57.

##### 2.2.3.2. Films Doped with Phenolic Compounds

HEC (0.2 g, ≥85.0%, Sigma-Aldrich) or PS was diluted in a vial containing 5 mL of deionized water, while the leaf extract (L) mass resulting from 5 mL methanol batch solid-liquid extraction was diluted in a different vial with 5 mL of methanol (≥99.9%, Sigma-Aldrich). Both solutions were heated in a 60 °C bath at 300 rpm for 60 min. The solutions were then mixed in the same vial and submitted to a 4 h ultrasounds bath (DT 106, Bandelin Electronic, Belin, Germany). The resulting solution was poured into a Teflon petri dish and left to dry in a cabinet dryer (Venticell, MMM Group) for 12 h at 60 °C. After 12 h, the resulting film was stored at selected atmospheres with different relative humidity values, as described in Section 2.2.3.1.

##### 2.2.3.3. Films Characterization

Scanning Electron Microscopy (SEM)

To evaluate the morphology of the developed films, cross-section and surface analysis were performed through scanning electron microscopy (SEM). Small samples of the films were cryofractured by immersion in liquid nitrogen, covered with a layer of Au-Pb and placed in a SEM device (S-2400, HITACHI, Tokyo, Japan). After preparation, the surface and cross-section of the samples were analyzed with 4 nm resolution, an electron beam intensity of 25 kV and 2.7 × 10^−5^ Pa ultimate vacuum. The magnifications studied were 400, 1000 and 2000×.

X-ray Diffraction (XRD)

To identify the crystallinity of the sample, X-ray diffraction (XRD) analysis was performed. The films’ samples were analyzed in an X-ray diffractometer (Miniflex, Rigaku, Tokyo, Japan), at room temperature, with a scanning range between 10–80°, using Cu X-Ray tube (30 kV/15 mA) as a radiation source.

Thermogravimetric Analysis (TGA)

To evaluate the thermal stability of the prepared films, thermogravimetric analysis (TGA) was carried out. TGA analysis was performed in a TA instrument (Labsys EVO Q50, Setaram, Caluire, France), in an argon atmosphere, with a temperature increase from 25 to 475 °C and a 10 °C/min heat range.

Fourier Transform Infrared (FTIR) Spectroscopy

To determine the interactions between the different film components, Fourier transform infrared (FTIR) spectroscopy was carried out. A square of the samples with dimensions of 1 × 1 cm was analyzed in a FTIR-ATR spectrometer (Spectrum Two, PerkinElmer, Waltham, MA, USA), with a wavelength range between 400–4000 cm^−1^ and 10 scans acquisition.

Contact Angel

To determine the films’ interaction with water, a film square with dimensions of 2 × 2 cm was cut and attached to the surface analyses of the goniometer (CAM 101, KSV Instruments Ltd., Helsinki, Finland). The analysis was performed using a drop of deionized water or glycerol deposited manually on the film surface with a small syringe. The measurements of 10 frames, with the intercalation of 1000 ms, was executed by a KSV software (CAM 2008, Laboratorytalk, Helsinki, Finland), immediately after the drop fell onto the film’s surface. The results were presented as an average of three measurements for each film.

Mechanical Properties

The mechanical properties of the films were analyzed using puncture tests. Samples were cut into squares of 3 × 3 cm and then cut into two identical triangles. These were analyzed using a texture analyzer (TA.XT plusC, Stable Micro Systems, Godalming, UK). The samples were fixed onto a flat platform with a 10 mm diameter hole and were punctured using a 2 mm diameter cylindrical probe, at a constant test speed (1 mm/s). For each film, three replicates were analyzed and the mean value of the normalized tensile strength (NTS) was determined. The tensile strength of each sample was calculated according to Equation (2) [26]:

σ = F/S_c_ (2)

where σ is the puncture tensile strength (Pa), F the force exerted by the probe (N), and S_c_ the probe cross-sectional area (m^2^).

For better accuracy in the comparison of the experimental results, the puncture tensile strength was normalized with the film thickness, measured with a micrometer (Elcometer 124, elcometer^®^, Manchester, UK), according to Equation (3):

σ_normalized_ = σ/l_film_ (3)

where σ_normalized_ is the NTS (MPa/mm) and l_film_ the film thickness (mm).

## 3. Results and Discussion

All the prepared samples were analyzed in triplicate. The results presented are the average of the three obtained values, except for Section 3.1 and Section 3.3.6 where means ± standard deviation (SD) of the three determinations are shown.

### 3.1. Phenolic Compounds Extracts Analysis

To determine the total phenolic content (TPC) and the antioxidant activity of the chemical compounds extracted from banana plant leaves, Folin Ciocalteau and DPPH methods were performed, respectively.

#### 3.1.1. Total Phenolic Content (TPC) Determination by the Folin Ciocalteau Method

**Figure 1:**
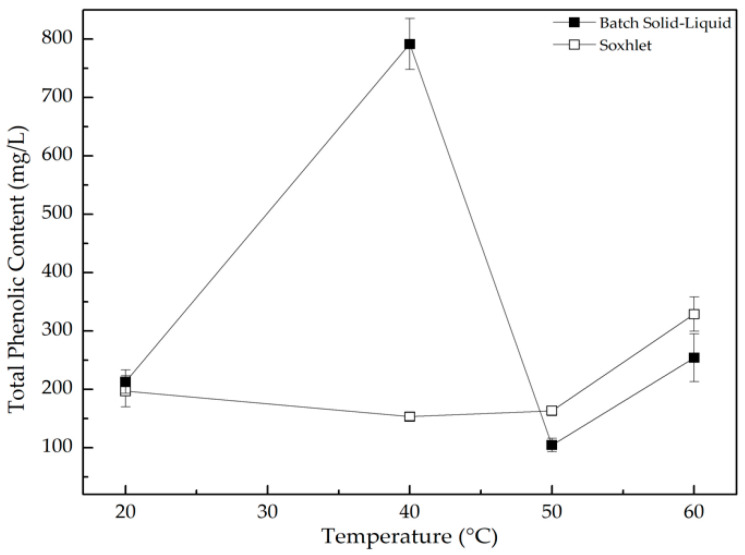
Total phenolic content results for batch solid-liquid (■) and Soxhlet (□), 3 days leaf extraction, using four different drying temperatures: 20, 40, 50 and 60 °C.

The total phenolic content (TPC) obtained using both extraction methods (batch solid-liquid and Soxhlet), are illustrated in Figure 1.

According to Dixon et al. [26] and Prathapan et al. [21], plants generally respond to drying temperature by increasing their phenolic content to repair the damaged tissue and by deactivating oxidative enzymes such as polyphenol oxidases. This statement was supported by the TPC increase from 20 to 40 °C that we observed in batch solid-liquid extraction. An increase in temperature can lead to the binding of phenolic compounds to other leaf components or to a chemical structure alteration, resulting in a phenolic content decrease, supporting the TPC decrease for temperatures higher than 40 °C [27]. In addition, in the experiments of Sagrin et al., the highest TPC was obtained for leaves dried at room temperature (2731.49 ± 14.41 mg), while in this work, the TPC obtained in the same conditions was 213.08 ± 20.08 mg [21]. The difference observed in the obtained values may be due to the fact that the banana plant samples used by Sagrin et al. [21] were from Malaysia and those used in this work were from Madeira, Portugal, thus being exposed to different environmental, cultivation and farming conditions [3].

#### 3.1.2. Antioxidant Activity Determination by DPPH Method

Using the results obtained from the Folin Ciocalteau method where the highest TPC (40 °C drying temperature) was the optimum, the antioxidant activity of the banana plant leaf extracts was measured. The results were 91.7 ± 0.5% and 82.8 ± 1.6% for batch solid-liquid and Soxhlet extraction methods, respectively. The highest degree of antioxidant activity was obtained with the batch solid-liquid extraction method. As expected, the high temperature used to evaporate the solvent in Soxhlet extraction led to degradation of the antioxidant compounds present in the leaves. This resulted in a lower value than the batch solid-liquid extraction method [21].

### 3.2. Cellulose Extraction and Analysis

Cellulose was extracted from 1 g of banana plant pseudostem, resulting in 0.073 g of cellulose. To confirm the cellulose extraction from banana plant pseudostem, 5 mg of the extract was exposed to an acidic digestion which allows the hydrolysis of cellulose into glucose. The glucose was then quantified at the retention time of 10.7 min using HPLC with an extraction yield of 7.3% (g cellulose/g pseudostem). This proved that the cellulose extraction was successful.

### 3.3. Films Analysis

The films are illustrated in Figure S1—Supplementary Material. All the prepared films were analyzed, except those where the leaf extracts were mixed with commercial hydroxyethyl cellulose (HEC + L) and where the banana plant pseudostem’s cellulose with the leaf extracts (PS + L) had been exposed to 0.843 a_w_.

#### 3.3.1. Scanning Electron Microscopy (SEM)

**Figure 2:**
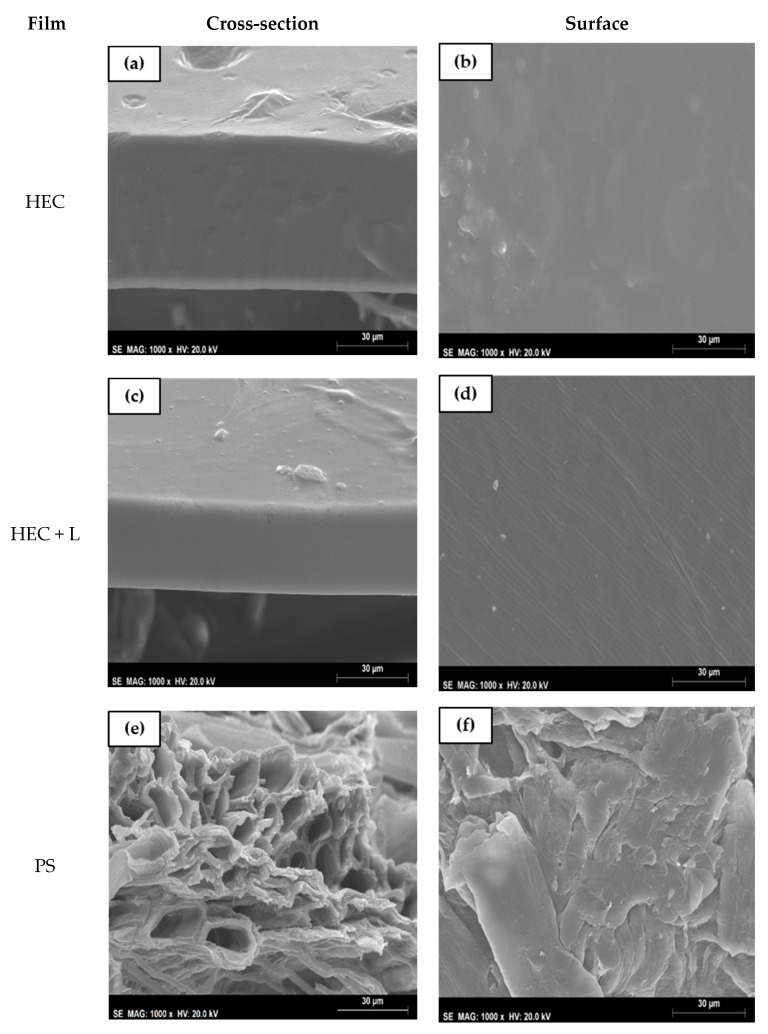

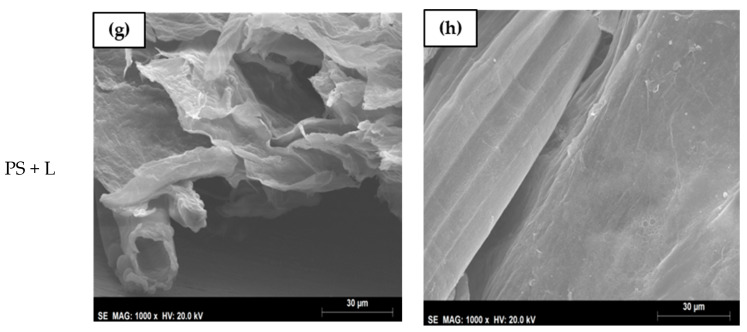
SEM cross section and surface images for HEC (**a**–**d**)—and PS films (**e**–**h**)—exposed to 0.577 a_w_, with 1000× magnification.

The analyzed SEM images are illustrated in Figure 2 (complementary to Figures S2 and S3—Supplementary Material). The films composed of HEC evidenced a homogeneous cross-section and surface without agglomerates.

The HEC + L films exposed to 0.577 and 0.753 a_w_, presented voids despite the homogenization process of the matrice constituents. These voids could have been introduced in the liquid inadvertently during the mixing process.

The PS and PS + L films had a rough surface and an entangled network of nanosized and polydisperse bundles, resulting in a non-homogeneous dispersal of the fibers in the film. This could be due to the low yield of cellulose extraction and the presence of other unidentified compounds which prevented better packaging of the fibers. In a porous film, this issue could be improved by using cross-linkers [28,29]. In addition, the PS film’s surface had small granules which are probably lignin polycondensate and deposits of inorganic minerals [29].

#### 3.3.2. X-ray Diffraction (XRD)

**Figure 3:**
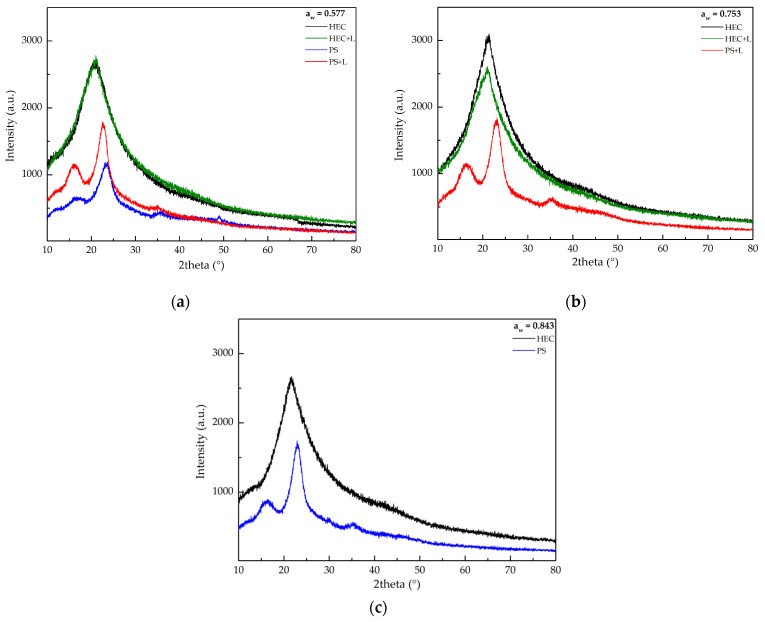
X-ray Diffraction results for HEC and PS films analysis exposed to (**a**) 0.577; (**b**) 0.753; (**c**) 0.843 a_w_.

The XRD results are illustrated in Figure 3. The peak observed at around 13° belongs to the glass substrate, as reported by Lim et al. [25]. This was clearly identified in PS and PS+L films. The other peak was observed at around 21.3°, in all the HEC films, which was also detected in earlier HEC reports [27].

PS and PS + L films evidenced crystalline peaks around 16°, 22° and 35°, which are typical from cellulose due to its hydrogen bonding interactions and the Van der Waals forces between adjacent molecules, as reported by Meng et al. [29].

These results show that the analyzed films are generally amorphous, since the peaks in the XRD spectrum have a low intensity, and it is possible to observe that the crystallinity of the samples is relatively higher for HEC films than for PS films.

#### 3.3.3. Thermogravimetric Analysis (TGA)

**Figure 4:**
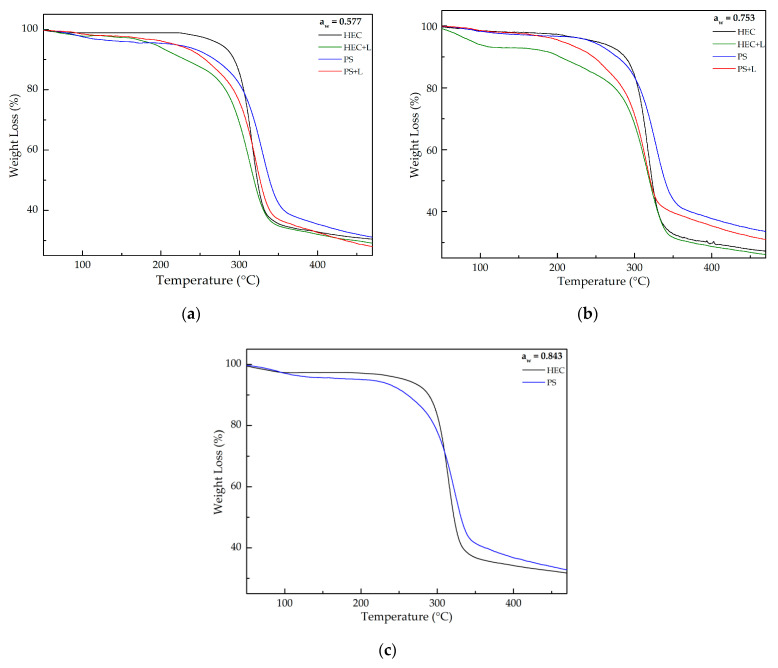
Weight loss as a function of temperature for the prepared films exposed to (**a**) 0.577; (**b**) 0.753; (**c**) 0.843 a_w_.

Figure 4 shows how the films’ components were degraded at different temperature ranges and Table S2—Supplementary Material charts the respective weight loss. The weight loss percentage was determined by comparing the initial and final weight loss.

It is possible to identify two degradation steps in the prepared films. The first weight loss, which can be observed in all the analyzed films, occurred at around 100 °C due to the evaporation of the water absorbed by the films. The second weight loss, at around 300 and 205 °C, was caused by the HEC and PS cellulose degradation processes such as depolymerization and dehydration of glycosyl units, respectively [25,30]. In both cases, HEC + L presented the lowest thermal stability when compared with PS films. It was not possible to identify a characteristic weight loss for the phenolic compounds present in the HEC + L and PS + L films. By analyzing Table S2 it was not possible to directly correlate variations in the a_w_ values with the thermal stability of the films.

#### 3.3.4. Fourier Transform Infrared (FTIR) Spectroscopy

**Figure 5:**
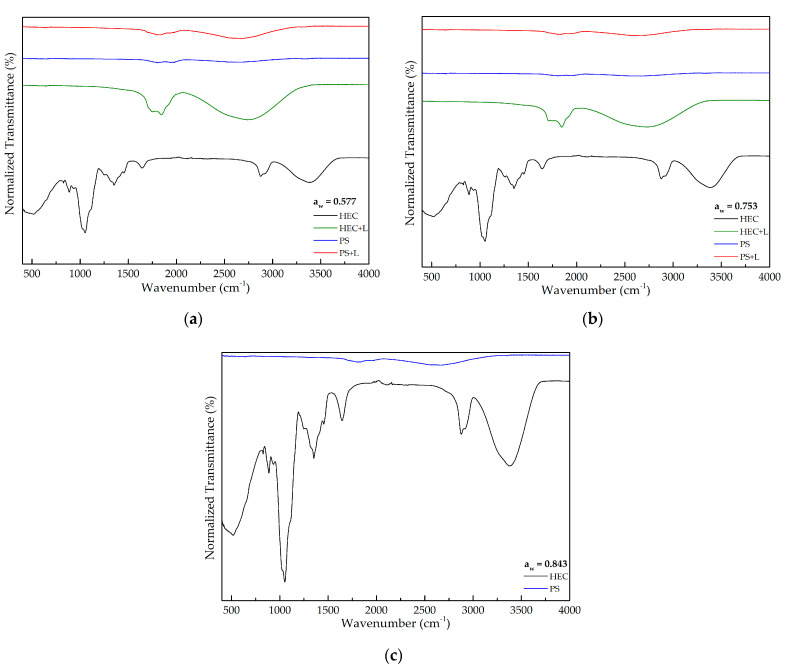
FTIR spectra for HEC and PS films exposed to (**a**) 0.577; (**b**) 0.753; (**c**) 0.843 a_w_.

Analyzing the peaks for HEC films (Figure 5), 1066 cm^−1^ peak can be attributed to the primary alcohol O–H bonding; the 1115 cm^−1^ peak the C–O–C bonding; and the 1385 cm^−1^ peak the C–H bonding. At around 1649 cm^−1^, the H–O–H bond is shown to be broader for HEC films. The peaks around 2337 cm^−1^ and 2362 cm^−1^ could be associated with the atmospheric CO_2_; 2893 and 2927 cm^−1^ were due to C–H symmetrical stretch; and the broad peak at 3340 cm^−1^ represents hydrogen bonding interactions with O–H stretching vibration. Since there are significant differences between the peaks for HEC and PS films it may indicate that the cellulose extracted was not entirely pure and may have included other unidentified compounds.

Regarding HEC films, it is possible to observe the intensity increase of the peaks around 1066 cm^−1^ (primary alcohol O–H bonding), 1649 cm^−1^ (H–O–H bond) and 3340 cm^−1^ (hydrogen bonding interactions with O–H stretching vibration) with the increase of a_w_, being caused by higher water content.

Comparing HEC and HEC + L films, the variation of the peaks caused by the interaction between the phenolic compounds and the other compounds present on the film can be observed. The peaks in HEC + L films around 1700 and 2700 cm^−1^ could be explained by the stretching of the carbonyl group (C=O) and the presence of CH_2_ symmetric stretching, respectively, in the phenolic compounds [31].

The presence of lignin is indicated by peaks at 761 cm^−1^ for C–H deformations, 1238 cm^−1^ for guaiacyl ring breathing with C=O stretching, 1525 cm^−1^ for aromatic ring vibrations, 1731 cm^−1^ for carboxyl group’s ester linkage of *p*-coumaric and ferulic acid or for acetyl and ironic ester groups of hemicellulose [30]. Since these peaks were not observed in the PS films we can deduce that the lignin and hemicellulose had been successfully removed during cellulose extraction, as intended.

#### 3.3.5. Contact Angle

The contact angle formed between the films surface and a drop of liquid (deionized water or glycerol) was determined. The hydrophobicity of the film to the solvent used (deionized water) increases with the increase of the contact angle.

The contact angles obtained with deionized water were impossible to determine, due to the immediate absorption of the drop of deionized water by the surface of the films, showing that all the analyzed films were hydrophilic. To quantify the contact angles, an organic solvent (glycerol) was used.

The films where the contact angle was possible to measure were: PS when exposed to 0.577 a_w_ (91.31 ± 0.33°), 0.753 a_w_ (104.49 ± 0.26°) and 0.843 a_w_ (114.37 ± 1.22°) and PS+L when exposed to 0.577 a_w_ (981.80 ± 7.63°) and 0.753 a_w_ (100.36 ± 4.41°).

Since an organic solvent was used, a film is considered hydrophilic when the contact angle is higher than 90° and hydrophobic when it is lower than 90°. Taking this into account, the most hydrophilic film was the one with the highest contact angle – PS at 0.843 a_w_ (114.37 ± 1.22°)—and the less hydrophilic film was PS at 0.577 a_w_ (91.31 ± 0.33°). These results prove the increase of hydrophilicity with the increase of a_w_ that the films were exposed to. In the case of the films doped with phenolic compounds, there was almost no variation between the analyzed water activities (98.8 ± 7.63° for 0.577 a_w_ and 100.36 ± 4.41° for 0.753 a_w_).

#### 3.3.6. Mechanical Properties

The obtained thickness and normalized tensile strength results are included in Table 1. The highest values of NTS were achieved, in general, for HEC films when exposed to 0.577 a_w._ It was possible to observe the increase of the NTS as the humidity decreased, showing a higher degree of force needed to break the films. The lower relative humidity improves the rigidity of the films. The higher rigidity prevents deformation (elongation) before breaking. This fact may be attributed to the plasticizer effect of water molecules adsorbed by the structures, which tends to be higher when exposed to high relative humidity values.

Comparing HEC and PS films, the NTS for PS films was considerably inferior. They lacked robustness due to the lower packing degree of their fibers—caused by the low cellulose content—as shown in SEM results. These results could be improved by the addition of plasticizers to the PS films, as achieved by Nataraj et al. [32].

## 4. Conclusions

This work aimed to develop polymeric films based on banana plant residues which otherwise have no economic value but have a detrimental impact on the environment when they are disposed of in rivers or in the countryside. The films were prepared with cellulose extracted from the banana plant pseudostem or commercial HEC, doped with phenolic compounds extracted from the banana plant leaves. The highest phenolic content (791.74 ± 43.75 mg/L) was achieved by batch solid-liquid extraction, with 40 °C leaves drying temperature, showing the potential of these residues for phenolics extraction. SEM analysis showed that the films prepared with HEC manifested a homogeneous dispersion of the matrice constituents and a dense structure, while the PS films presented a porous structure, due to the lack of fibers packaging caused by the low yield of cellulose extraction. FTIR results confirm the incorporation of phenolic compounds in HEC films and the presence of non-identified compounds in PS films, as well as the successful removal of lignin and hemicellulose during the cellulose extraction procedure. Mechanical properties assays show the increase of NTS as the a_w_ decreases for HEC films and the low NTS for PS films caused by the low fibers packaging, which can be improved using plasticizers. Despite the need for the cellulose extraction optimization it can be observed that PS films showed promise for films production in areas such as wound dressing or food packaging, due to its similarities to HEC films in terms of XRD (amorphous), TGA (high thermal stability) and contact angle (hydrophilic) characterization.

## Figures and Tables

**Table 1:** Thickness and normalized tensile strength results for the analyzed films, exposed to 0.577, 0.753 and 0.843 a_w_.

Film	Thickness (μm)			Normalized Tensile Strength (MPa/mm)		
	0.577 a_w_	0.753 a_w_	0.843 a_w_	0.577 a_w_	0.753 a_w_	0.843 a_w_
HEC	116.00	117.33	101.33	37.10 ± 0.04	12.47 ± 2.78	13.44 ± 0.65
HEC + L	56.67	66.00	-	52.62 ± 0.50	6.52 ± 1.90	-
PS	112.00	157.33	70.67	2.13 ± 0.03	4.27 ± 0.09	2.87 ± 0.13
PS + L	82.00	120.00	-	1.89 ± 0.10	1.09 ± 0.07	-

## Data Availability

The data presented in this study are available on request from the corresponding author.

## Supplementary Materials

The following are available online at https://www.mdpi.com/2073-4360/13/5/843/s1, Figure S1: Films prepared: (a) HEC at 0.577, 0.753 and 0.843 a_w_; (b) HEC + L at 0.577 and 0.753 a_w_; (c) PS at 0.577, 0.753 and 0.843 a_w_; (d) PS + L at 0.577 and 0.753 a_w_; Figure S2. Scanning Electron Microscopy (SEM) analysis results for HEC (a–d) and PS (e–h) films exposed to 0.753 aw, with 1000× magnification; Figure S3: SEM analysis results for HEC and PS films exposed to 0.843 a_w_, with 1000× magnification; Table S1: Samples preparation for DPPH Method; Table S2: Weight loss and respective temperature range, resultant from TGA analysis of the films exposed to 0.577, 0.753 and 0.843 a_w_.
