# Supplementary material for: Rendering Banana Plant Residues into a Potentially Commercial Byproduct by Doping Cellulose Films with Phenolic Compounds
Source: Polymers (Basel). 2021 Mar 9;13(5):843. doi: 10.3390/polym13050843 (PMC7967194; doi:10.3390/polym13050843)
Supplement: Supplementary file 1 [file polymers-13-00843-s001.pdf]

polymers

Supplementary Materials: Rendering Banana Plant Residues into a Potentially Commercial By-Product by Doping Cellulose Films with Phenolic Compounds

Rosa E. A. Nascimento, Joana Monte, Mafalda Cadima, Vítor D. Alves and Luísa A. Neves

Table S1. Samples preparation for DPPH Method.

	Dilution	$V_{extract}$ (mL)	$V_{methanol}$ (mL)	$V_{working\ solution}$ (mL)
White	-	-	0.5	3.5
Extract solution	1:1	0.5	-	3.5
	1:2	0.25	0.25	3.5
Control	1:1	0.5	3.5	-
	1:2	0.25	3.75	-

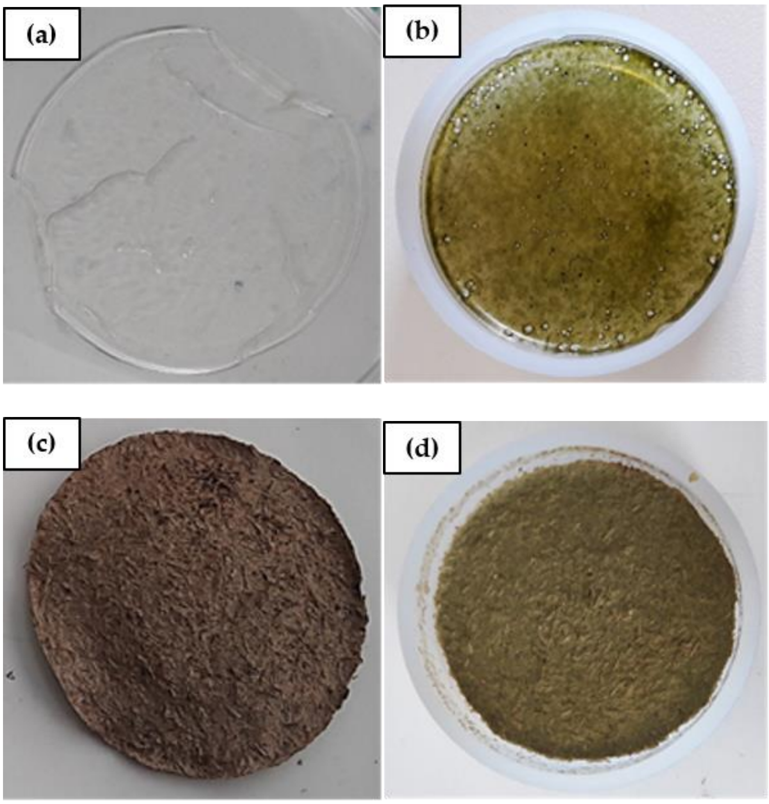

Figure S1. Films prepared: **(a)** HEC at 0.577, 0.753 and 0.843 a_w; **(b)** HEC+L at 0.577 and 0.753 a_w; **(c)** PS at 0.577, 0.753 and 0.843 a_w; **(d)** PS+L at 0.577 and 0.753 a_w.

**Film**	**Cross-section**	**Surface**
HEC	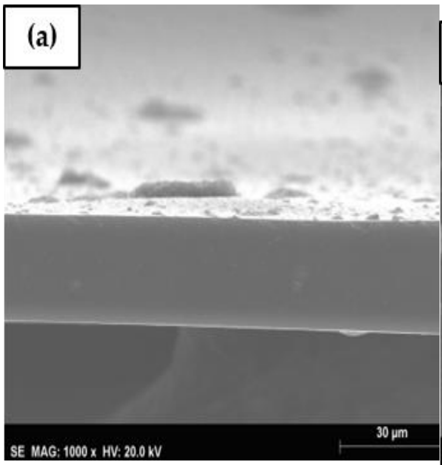	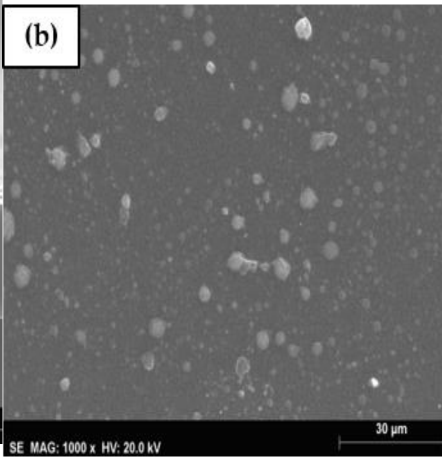
HEC+L	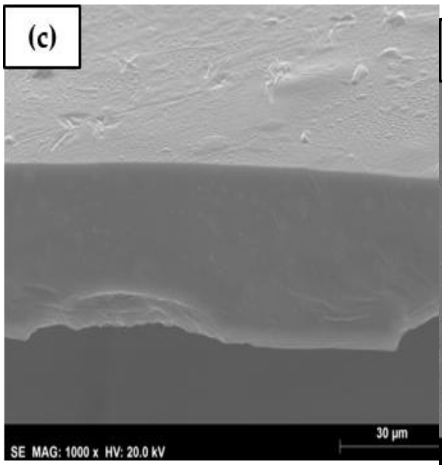	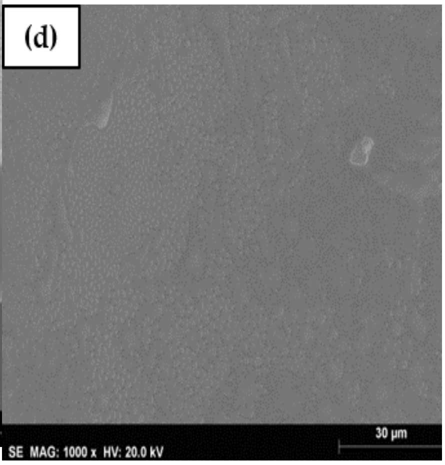
PS	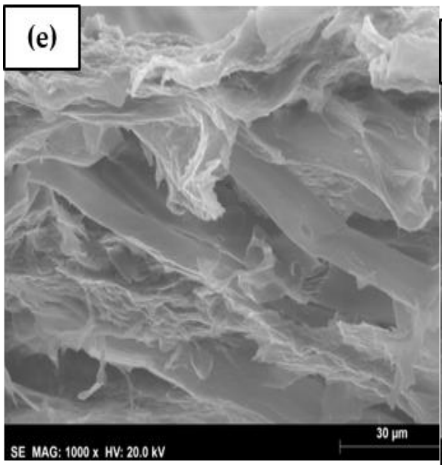	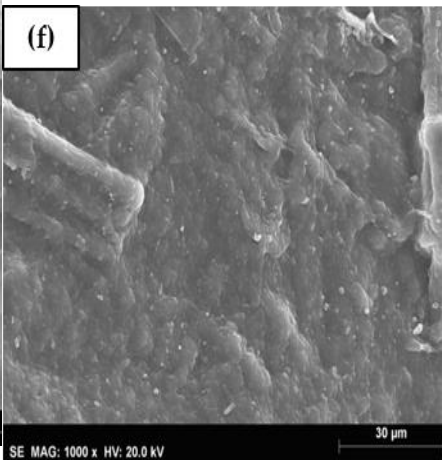

PS+L

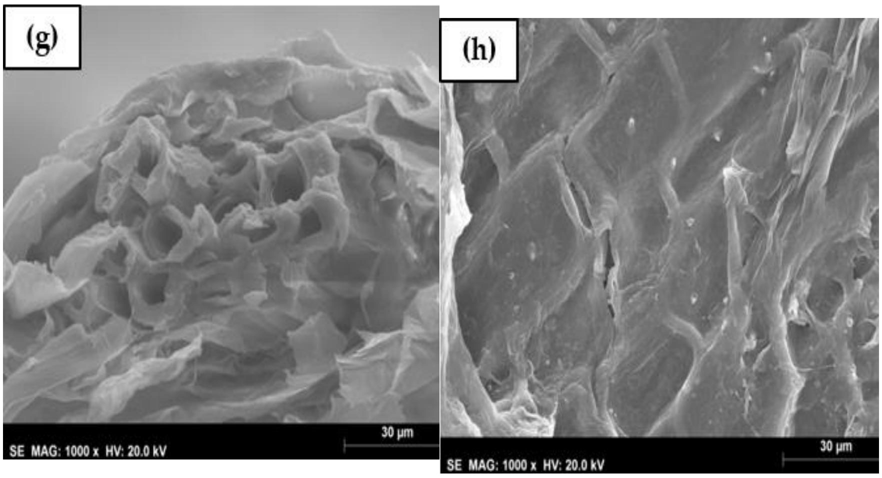

Figure S2. Scanning Electron Microscopy (SEM) analysis results for HEC (**a–d**) and PS (**e–h**) films exposed to 0.753 aw, with 1000 × magnification.

**Film**	**Cross-section**	**Surface**
HEC		
PS		

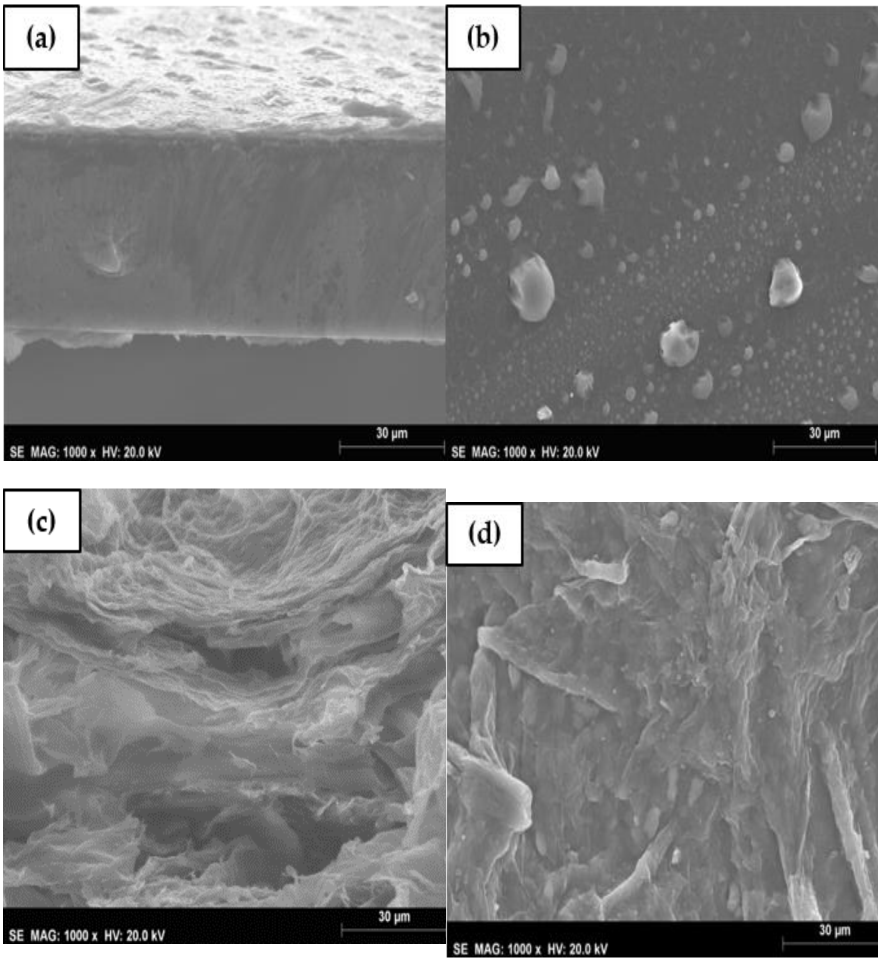

Figure S3. SEM analysis results for HEC(**a, b)** and PS (**c, d)** films exposed to 0.843 a_w, with 1000 × magnification.

Table S2. Weight loss and respective temperature range, resultant from thermogravimetric analysis (TGA) of the films exposed to 0.577, 0.753 and 0.843 a_w.

Film 0.577 a_w	Total weight loss (%)	Temperature range (°C)	Film 0.753 a_w	Total weight loss (%)	Temperature range (°C)	Film 0.843 a_w	Total weight loss (%)	Temperature range (°C)
HEC	1.14<br>70.06	49.72–115.95<br>176.26–466.04	HEC	71.69	46.37–443.42	HEC	2.09<br>67.49	46.90–132.06<br>172.42–464.33
HEC +L	2.72<br>70.9	32.16–126.29<br>126.39–456.38	HEC +L	7.22<br>73.47	32.55–139.54<br>140.19–455.15	-	-<br>-	-<br>-
PS	4.03<br>68.17	35.29–164.38<br>192.6–463.86	PS	3.45<br>66.32	33.05–180.96<br>181.42–462.35	PS	4.32<br>66.93	47.88–176.97<br>176.97–472.71
PS+L	2.09<br>71.37	47.35–152.38<br>152.64–463.66	PS+L	2.41<br>68.85	49.67–151.13<br>150.08–466.91	-	-<br>-	-<br>-
